# Maternal and Fetal Outcomes of Pregnant Women Infected with Coronavirus Based on Tracking the Results of 90-Days Data in Hazrat -E- Rasoul Akram Hospital, Iran University of Medical Sciences

**DOI:** 10.30476/BEAT.2021.90434.1254

**Published:** 2021-07

**Authors:** Shahla Chaichian, Abolfazl Mehdizadehkashi, Shahla Mirgaloybayat, Neda Hashemi, Farahnaz Farzaneh, Roya Derakhshan, Samaneh Rokhgireh

**Affiliations:** 1 *Pars Advanced and Minimally Invasive Medical Manners Research Center, Pars Hospital, Iran University of Medical Sciences, Tehran, Iran*; 2 *Endometriosis Research Center, Iran University of Medical Sciences (IUMS), Tehran, Iran*

**Keywords:** COVID-19, Pregnancy, Maternal death, Adverse events, Outcome, Iran

## Abstract

**Objective:**

To evaluate the maternal and fetal outcomes of COVID-19 up to three months after the delivery in pregnant women.

**Methods:**

This case series study was conducted on all pregnant women with COVID-19 hospitalized in Hazrat -E- Rasoul Akram Hospital, Tehran, Iran from March 8, 2020 to December 28, 2020. Data were included maternal age and gestational age (GA) which presenting signs and symptoms were collected at hospital admission. To confirm COVID-19 diagnosis, high-resolution computed tomography (HRCT) or reverse transcription-polymerase chain reaction (RT-PCR) tests were conducted. Both the mothers and the newborns were followed up to three months after delivery.

**Results:**

Fourteen pregnant women with the median age of 31.5 were enrolled. HRCT was done in twelve mothers (85.7%), and eleven mothers (78.6%) were evaluated via RT-PCR; four of them (36.36%) were positive. Two mothers (14.28%) were admitted to ICU. The cesarean section (C/S) was done following fetal distress in only three mothers due to their concerns of vertical transmission. Two mothers were admitted to the intensive care unit (ICU), and one of them died of pneumomediastinum. Fortunately, no neonatal death was reported three months after the delivery.

**Conclusion:**

COVID-19 affects mothers more in the last trimester of the pregnancy. Although no fetal death was reported in the recent study, physicians should closely monitor pregnant women to reduce the adverse event .

## Introduction

Coronavirus disease (COVID-19) is a highly contagious respiratory infection that first reported in Wuhan, China, and became a global pandemic in about three months [1-3]. Since early March, Iran has been involved too. According to the latest official statistics, more than 1,600,000 confirmed cases and about 5900 deaths were reported in Iran [4]. The low experience of dealing with patients and specific conditions of pregnant women with COVID-19 requires a careful investigation and recognition of clinical and paraclinical consequences to identify and manage these patients [5].

Although the treatment of non-pregnant patients is based on the world health organization’s or national protocols, the lack of long-term information, the small number of pregnant cases, and discrepancies between the outcome of pregnant women and other patients have caused the inability to make proper decisions [6-9]. Among the first reported studies, a case series conducted by Zhu* et al*. examined the maternal and neonatal symptoms and outcomes of 9 pregnant women with COVID-19. Fetal distress, preterm labor, and liver-induced thrombocytopenia have been reported, but none of the newborns were infected with COVID-19 [7, 9]. However, the possible adverse effects of diagnostic and therapeutic interventions have not been evaluated in several published articles yet such as computed tomography (CT), steroids and chloroquine, and other prescribed drugs on the fetal status and laboratory changes in pregnant women with COVID-19. Therefore, in this cross-sectional study, we aimed to evaluate the maternal and fetal outcomes of COVID-19 up to three months after the delivery. 

## Materials and Methods

Study Population

This case series study was done with the definite diagnosis of COVID-19 in all pregnant women admitted to Hazrat -E- Rasoul Akram Hospital, Iran University of Medical Sciences (IUMS), Tehran, Iran from March 8, 2020 to December 28, 2020. All the pregnant women were included with the definite diagnosis of COVID-19 regardless of their gestational age (GA), having COVID-19-related signs and symptoms according to the guideline provided by the Iranian Ministry of Health and Education (MOHME) [10]. Definite cases were those with positive reverse-transcription polymerase chain reaction (RT-PCR) test and/or with typical features of COVID-19 on high-resolution CT (HRCT) images based on the Radiological Society of North America (RSNA) criteria [11]. Before the enrollment, we asked patients to assign a written informed consent to engage in this case series study. The exclusion criteria were pregnant women without a definite diagnosis of COVID-19 cases, out-patient mothers, or unwillingness to participate in the study.

Study Design

Admission evaluations: Patients’ demographic data, such as age, gestational age (reported as a week day/7 [w d/7]), their chief complaints regarding COVID-19 related signs and symptoms were collected at the time of hospital admission. RT-PCR test and/or HRCT images were taken to confirm the diagnosis on patients’ consent. Laboratory findings includes white blood count (WBC), lymphocyte percentage (Lymph%), blood group, the serum level of aspartate aminotransferase (AST), and alanine aminotransferase (ALT) were extracted from the university’s comprehensive database. 

 If needed, patients were admitted according to the national protocol in the specialized ward or the intensive care unit (ICU). Midwifery adverse events were investigated which were defined as any of the following: labor pain, vaginal bleeding, premature rupture of membrane (PROM), and fetal movements, and primary fetal heart monitoring was performed in the second and third trimesters. These parameters were daily recorded during hospital admission. However, the data were not shown. 

In-patients’ care: Previously mentioned lab data, clinical signs, and symptoms were assessed persistently during the hospital admission at the infectious discretion and internal services. Routine treatment includes antiviral medications, antibiotic drugs and chloroquine was provided for all the patients according to the national protocol under the control of an infectious disease specialist. we terminated a pregnancy in situations such as fetal distress or any other life-threatening maternal or fetal conditions. Moreover, the mode of delivery (MOD) was chosen based on fetomaternal clinical and obstetric states. After termination, newborns’ information includes Apgar scores, weight were needed for neonatal ICU (INCU) admission. Diagnosis to delivery interval (DDI) was calculated by counting the days between the first hospital admission and the delivery time.

In- or Out-patients follow-ups: We could discharged the patients if all of the following criteria were met: O_2_ saturation (O_2_ sat) ≥93% without oxygen supplements, being afebrile for at least two days and the chest radiological findings’ improvements. Treatments were continued after discharge in accordance to the infectious disease specialist’s opinion. In the absence of emergency midwifery conditions or the respiratory symptoms’ return, follow-up visits were performed in the 2^nd^ and 4^th^ weeks after discharge. In this study, the fate of mother and fetus was followed up by telephone for three months, while sending data to the University’s database which is a detailed examination of maternal and fetal outcomes and treatment management based on the results of out-patient and in-patient care of pregnant women was investigated by the end of third months.

Statistical Analysis 

The gathered data were analyzed by using the Statistical Package for Social Sciences version 20 (SPSS Inc., Chicago, IL, US) software. Median and interquartile range (IQR) were used to describe quantitative variables, and the qualitative ones were reported using the frequencies and percentages.

## Results

In the recent survey, 14 pregnant women were enrolled with the median age of 31.5 (IQR: 27.75-38.25) ranged between 26-43 years-old. HRCT was done in 12 mothers (85.7%), and the 2 (14.3%) did not assign the written informed consents for HRCT. Eleven mothers (78.6%) were evaluated via RT-PCR that 4 of them (36.36%) were positive (Table 1).

**Table 1:** Demographic and clinical feature of enrolled pregnant women (n=14)

**Patients’ NO.**	**Age (y/o** ^c^ **)**	**AST** ^a^ ** (U/L)**	**ALT** ^b^ ** (U/L)**	**LYMP** ^d^ ** (%)**	**WBC** ^e^ ** (10** ^6^ **/L)**		**CRP** ^f^ ** (mg/L)**		**HRCT** ^g^	**RT-PCR** ^h^	**CC** ^i^	**BG** ^j^	**DDI** ^k^ ** (d)**	**MOD** ^l^	**GA** ^m^ * (w d/7)	**GA** **(d)**	**Steroids** ^**^	**Antibiotics**	**Antiviral**	**Chloroquine**	**HFO**_2_^n^	**ICU** ^o^	**Outcome** ^***^
1	33.0	71.0	52.0	N/A	8.2		48.0		Typical	-	Myalgia	O+	21.0	C/S^t^	36 3/7	255.0	-	+	+	+	-	-	Alive
2	30.0	152.0	116.0	10.0	9.5		49.0		Typical	-	Myalgia	B+	46.0	C/S^t^	31 5/7	222.0	+	+	+	-	-	-	Alive
3	28.0	30.0	18.0	15.0	17.3		N/A		Typical	-	Myalgia	B+	42.0	C/S^t^	32 0/7	224.0	-	+	+	+	-	-	Alive
4	28.0	37.0	24.0	1.3	8.3		N/A		Typical	N/A^p^	Myalgia	A+	44.0	C/S^t^	32 3/7	227.0	-	-	+	+	-	-	Alive
5	30.0	N/A	N/A	15.7	8.7		N/A		Typical	-	-	B+	105.0	C/S^t^	24 6/7	174.0	-	+	-	-	-	-	Alive
6	43.0	28.0	29.0	N/A	6.9		N/A		Typical	N/A^p^	Cough	O+	1.0	NVD^s^	39 5/7	278.0	-	-	-	-	-	-	Alive
7	36.0	N/A	N/A	N/A	N/A		N/A		Typical	N/A^p^	Cough	B+	14.0	C/S^t^	36 0/7	252.0	-	+	+	+	-	-	Alive
8	39.0	50.0	22.0	8.3	10.1		24.0		Typical	-	Cough	O+	7.0	C/S^t^	30 2/7	212.0	+	+	+	-	+	+	Alive
9	26.0	45.0	29.0	24.9	7.1		12.0		Typical	-	Fever	A+	8.0	C/S^t^	31 2/7	219.0	-	+	+	+	-	-	Alive
10	27.0	38.0	28.0	12.9	11.7		N/A		N/A	+	Myalgia	O+	1.0	C/S^t^	37 1/7	260.0	-	+	-	-	-	-	Alive
11	26.0	N/A	N/A	4.6	17.3		N/A		N/A	+	Fever	A+	N/A	N/A	9 3/7	66.0	-	+	-	-	-	-	Alive
12	43.0	31.0	24.0	7.0	17.8		48.0		Typical	-	Myalgia	A+	49.0	C/S^t^	31 1/7	218.0	+	+	+	+	-	-	Alive
13	38.0	58.0	41.0	21.0	7.3		N/A		Typical	+	Fever	O+	3.0	C/S^t^	33 4/7	235.0	+	+	+	-	+	+	Dead
14	35.0	37.0	20.0	17.0	10.0	N/A		Normal		+	Cough	B+	17.0	NVD^s^	39 0/7	273.0	-	+	-	-	-	-	Alive

^a^AST: Aspartate Transaminase; ^b^ALT: Alanine Transaminase; ^c^y/o: year-old; ^d^LYMP: Lymphocyte; ^e^WBC: White Blood Cell; ^f^CRP: C-reactive Peptide; ^g^RCT: High Resolution Computed Tomography; ^h^RT-PCR: Reverse Transcription Polymerase Chain Reaction; ^i^CC: Chief complain; ^j^BG: Blood Group; ^k^DDI: Diagnosis to Delivery Interval; ^l^MOD: Mode of Delivery; ^m^GA (w d/7): Gestational Age (week day); ^n^HFO_2:_ High Flow O_2_; ^o^ICU: Intensive Care Unit; ^p^N/A: Not Available; ^q^+, Positive; -, ^r^Negative; ^s^NVD: Normal Vaginal Delivery; ^t^C/S: Caesarean section; *Gestational Age at the time of delivery; ** Medical treatment was performed based on the Iranian national guideline COVID 19 on pregnant women; ***Outcome defined as the maternal fate three months after delivery.

The youngest mothers were two 26-years-old women with GA of 9 3/7 and 31 2/7. The former was still pregnant when this case series was being reported. She presented to our center, and her chief complaint was fever accompanied by headache, body pain, and blood work-ups revealed leukocytosis (17.3×10^6^/L) and lymphopenia (4.6%). The nasopharyngeal RT-PCR test confirmed the COVID-19 diagnosis, and we did not perform HRCT to reduce the unnecessary radiation dose exposure. She was admitted to our specialized COVID-19 ward without the development of hypoxia and dyspnea. She was discharged from our hospital and was alive until the present time.

The oldest mother was 43 years-old woman with the GA of 39 5/7, brought to the Hazrat -E- Rasoul Akram Hospital with the cough. She refused to receive a RT-PCR test, and the typical features of COVID-19 were detected on HRCT images. A day after hospital admission, she developed dyspnea and fetal distress, therefore, normal vaginal delivery was (NVD) done. Both the mother and the baby were alive until three months after the delivery.

Two mothers (14.28%) were admitted to ICU and treated with High-flow oxygen (HF O_2_). If symptoms includes non-invasive refractory hypoxia, loss of consciousness, hemodynamic instability, respiratory fatigue and hypercapnia persist, patients were admitted to the ICU according to the Iranian National Guideline to Diagnosis and Treatment of Covid Disease in Pregnancy [12]. The cesarean section (C/S) was done following O_2_ saturation reduction and fetal distress. The first mother was a 39 years-old female with the GA of 30 2/7 and had dry coughs at the time of admission. She was intubated in the ICU for seven days after C/S and was treated with Atazanavir, Favipiravir, Chloroquine, and broad-spectrum antibiotic and Steroid, medical treatment was performed based on the Iranian national guideline COVID 19 on pregnant women and discharged after complete recovery. The second mother was in her late 40s with the GA of 33 4/7 and intubated in the ICU. Unfortunately, despite receiving the previously mentioned drugs, she died of pneumomediastinum on the second day after C/S. Both infants were discharged from the NICU in good general condition.

As shown in Table 2, 13 neonates were born, and the 11^th^ mother was still pregnant. The median of GA at the time of hospital admission was 32 2.5/7 (IQR: 30.75 0.75/7- 36.25 4.25/7). The median Apgar score was nine and ten after one and five minutes, respectively. The former had an IQR of 8-9.5, while the last had an IQR of 9-10. All born neonates had negative RT-PCR tests, and only two out of 13^th^ born babies (15.38%) were admitted to the NICU. Happily, all neonates (100%) were in proper clinical conditions.

**Table 2:** Clinical feature and COVID-19 test in the corresponding neonates

**Child No.**	**BWt** ^a ^ **(gr** ^b^ **)**	**GA** ^c^ ** (w d/7 )**	**GA (d)**	**AP5** ^e^	**AP1** ^d^	**NRT-PCR** ^f^	**NICU** ^g^
1	3010	36 3/7	255.0	10	9	-	-
2	3470	31 5/7	222.0	10	9	-	-
3	3700	32 0/7	224.0	9	10	-	-
4	2780	32 3/7	227.0	10	9	-	-
5	2950	24 6/7	174.0	9	10	-	-
6	3600	39 5/7	278.0	10	9	-	-
7	2800	36 0/7	252.0	10	9	-	-
8	1900	30 2/7	212.0	7	3	-	+
9	1600	31 2/7	219.0	9	7	-	+
10	3200	37 1/7	260.0	10	9	-	-
11	N/A	9 3/7	66.0	N/A	N/A	N/A	N/A
12	2200	31 1/7	218.0	9	10	-	-
13	2630	33 4/7	235.0	9	7	-	-
14	3020	39 0/7	273.0	10	9	-	-

^a^BWt: Birth Weight; ^b^gr: gram; ^c^GA (w d/7): Gestational Age ( week day/7); ^d^AP1: Apgar Score one minute after delivery; ^e^AP5: Apgar Score five minute after delivery; ^f^NRT-PCR: Neonatal Reverse Transcription Polymerase Chain Reaction; ^g^NICU: Neonatal Intensive Care Unite

## Discussion

In this study, 12 out of 14 pregnant women were in the third trimester, and the two were in the first and second trimester. None of the mothers had high blood pressure, diabetes, or cardiovascular disease (data were not shown). Two out of fourteen women under study were admitted to the intensive care unit, and one of them died. Two out of the 13 newborn babies were born through the NVD, and the rest were by the C/S. In a systematic review study conducted by Zaigham* et al*., on the pregnancy evaluated of 108 women and showed the superiority of third-trimester involvement as reported in our study [13]. Nakamura* et al*., believed that the increased mortality rate in pregnant mothers was partly due to the high rate of obesity and pre-eclampsia in the third trimester [14].

During this case series study, we confirmed the COVID-19 diagnosis via HRCT in 11 pregnant women (78.57%), and RT-PCR tests were positive in only 4 cases (27.43%). The 13^th^ mother had a positive RT-PCR test and typical feature of COVID-19 on HRCT images. Similar studies have emphasized the importance of CT findings compared to RT-PCR [8]. A review article of 7 studies conducted by Kucirka* et al*., indicated that the false-negative rate of the RT-PCR test varied according to the time trends of the disease; it ranged from 100% to 67% within the four days before the symptoms evolved. During the symptomatic phase, the median false-negative rate of the RT-PCR test showed a biphasic trend; it decreased from 38% (on the first day of being symptomatic) to 20% (during the eighth day) and then increased up to 66% on day 21 [15]. Therefore, we had to evaluate the patients via HRCT to confirm the COVID-19 diagnosis and assess the lung-involvement severity.

Overall, eleven neonates were born via C/S; three mothers gave birth to their babies via C/S due to their concerns after COVID-19, which led them not to cooperate in NVD. One other neonate was in the breech position, and for the other seven mothers, C/Ss were done due to the previous C/S. Studies from China report higher rates of C/S in mothers with Covid-19, while in Spain, in a cohort study, 53% of patients had NVDs, and 47% had a cesarean section [16]. Moreover, Cai *et al*., designed a systematic review study to determine whether the MOD could affect the COVID-19 vertical transmission between the mothers and their babies. They have concluded that the vertical transmission was not affected by the MOD, and the physicians must choose the proper MOD based on the obstetric indications and the disease severity [17]. Although, some case report studies showed the possibility of vertical transmission [18, 19].

Myalgia and fatigue were the most common symptoms among mothers at the time of hospitalization. However, in most studies, cough and fever were the first common symptoms [6]. In this study, two women had severe disease and resulted in intubation and the subsequent maternal death in one pregnant woman. As shown in the result section, pneumomediastinum was the leading cause of maternal death, and multiple studies have been conducted reporting the possible effect of COVID-19 in pneumomediastinum development [20-22]. Panahi* et al*., showed no differences in the COVID-19-related signs and symptoms between the pregnant and non-pregnant women which is inconsistent with our case series study [23]. This discrepancy might result from our low sample size and the case series nature of the study.

In contrast to the symptoms frequency’s similarities, the Center for Disease Control and Prevention (CDC) declared that COVID-19 had more severe clinical trends in pregnant women and resulted in higher hospitalization and ICU admission rates and the subsequent more severe adverse outcome [24].

## Conclusion

This study shows that most of the maternal involvement occurred during the third trimester of pregnancy, and despite the severe involvement, neonatal COVID-19 involvement or mortality was not reported. Although the pregnant women still need to be closely monitored to prevent and decrease the maternal and neonatal mortality rate and adverse events. Moreover, the COVID-19 diagnosis must have relied on patients’ clinical symptoms and lung CT findings due to the false-negative results of the RT-PCR test. 

## Ethical Approval:

This study was approved by the ethics committee of the Faculty of Medicine, Hazrat -E- Rasoul Akram Hospital, Iran University of Medical Sciences (IUMS), Tehran, Iran from March 8, 2020 to December 28, 2020 by ethics code PH.REC.1399.009.

## Funding Support:

None declared.
